# Plasma microRNA profiling: Exploring better biomarkers for lymphoma surveillance

**DOI:** 10.1371/journal.pone.0187722

**Published:** 2017-11-13

**Authors:** Drirh Khare, Neta Goldschmidt, Aya Bardugo, Devorah Gur-Wahnon, Iddo Z. Ben-Dov, Batia Avni

**Affiliations:** 1 Department of Hematology and Bone Marrow Transplantation & Cancer Immunotherapy, Hadassah-Hebrew University Medical Center, Jerusalem, Israel; 2 Laboratory of Medical Transcriptomics, Nephrology and Hypertension Services, Hadassah - Hebrew University Medical Center, Jerusalem, Israel; Universitat des Saarlandes, GERMANY

## Abstract

Early detection of relapsed lymphoma improves response and survival. Current tools lack power for detection of early relapse, while being cumbersome and expensive. We searched for sensitive biomarkers that precede clinical relapse, and serve for further studies on therapy response and relapse. We recruited 20 healthy adults, 14 diffuse large B-cell lymphoma (DLBCL) patients and 11 Hodgkin lymphoma (HL) patients at diagnosis. Using small-RNA sequencing we identified in DLBCL patients increased plasma levels of miR-124 and miR-532-5p, and decreased levels of miR-425, miR-141, miR-145, miR-197, miR-345, miR-424, miR-128 and miR-122. In the HL group, we identified miR-25, miR-30a/d, miR-26b, miR-182, miR-186, miR-140* and miR-125a to be up-regulated, while miR-23a, miR-122, miR-93 and miR-144 were down-regulated. Pathway analysis of potential mRNAs targets of these miRNA revealed in the DLBCL group potential up-regulation of STAT3, IL8, p13k/AKT and TGF-B signaling, and potential down-regulation of the PTEN and p53 pathways; while in the HL group we have found the cAMP-mediated pathway and p53 pathway to be potentially down-regulated. Survival analyses revealed that plasma levels of miR-20a/b, miR-93 and miR-106a/b were associated with higher mortality. In conclusion, we identified sets of dysregulated circulating miRNA that might serve as reliable biomarkers for relapsed lymphoma.

## Introduction

Lymphoid neoplasms are the fourth and fifth most common cancer and the sixth leading cause of cancer death in the United States and Canada [1, 2]. In 2016, lymphoid neoplasms were newly diagnosed in 136,960 patients in the United States. Hodgkin lymphomas make up 6% of all lymphoid neoplasms, and most are classical Hodgkin lymphomas (mainly nodular sclerosis subtype). Among the non-Hodgkin lymphoma (NHL) neoplasms, diffuse large B-cell lymphoma (DLBCL) is the most common subtype comprising about one-quarter of NHL neoplasms [2]. Diagnosis of lymphoma is based on surgical tumor biopsy and PET-CT is considered the standard imaging examination, both for staging and response evaluation. In the follow-up setting, PET is not considered a good tool due to high incidence of false-positive results [3]. The false-positive rate with PET scans is greater than 20%, leading to unnecessary investigations, radiation exposure, biopsies, expense, and patient anxiety. Currently, follow-up scans are prompted by clinical indications. Better laboratory and clinical markers are needed for following residual masses and detecting early progression or relapse [4, 5].

MicroRNAs (miRNAs) are endogenous short (~22-nucleotides) non-protein-coding regulatory RNA molecules. The biochemical association of miRNA with mRNA regulates gene expression at the posttranscriptional level by either suppressing stability or translation of the mRNA, or both of these processes. Single miRNA species can control different target mRNAs and consequently influence production of multiple proteins. Conversely, multiple miRNAs can often synchronously target the same mRNA and jointly control expression of the particular gene product. miRNAs have the potential to regulate multiple functionally related genes involved in a specific biological pathway. The cooperative miRNA interactions with the target mRNAs can influence a variety of critical biological programs such as cell division, apoptosis, differentiation, development, senescence, metabolism, control of hematopoiesis and tumorigenesis. Numerous miRNAs have become candidates for diagnostic and prognostic biomarkers and targets for cancer therapeutic intervention. In light of miRNAs’ potential as diagnostic markers in cancer [6] there is increasing interest in the possible role for miRNAs as markers for both B-cell differentiation stage and malignant transformation. It has been shown that miRNA expression patterns can characterize stages of human B-cell differentiation. A large number of miRNA signatures characterizing lymphomas were identified, and the role of miRNAs in the development, classification and in the regulation of target genes is under intensive investigation [7–11].

Recently, miRNAs have been identified outside the cells in a range of body fluids. In the circulation they are commonly found enclosed in extracellular vesicles (EVs), bound to lipoproteins, or complexed with Argonaute proteins [12]. Chim et al and Williams et al identified the expression of placental miRNAs in the circulation of pregnant women [13, 14]. Lawrie et al reported the augmented level of different miRNAs analyzed by qRT-PCR such as miR-21, miR-210, and miR-155 in serum of B cell lymphoma patients [15]. Several studies presented circulating miRNAs as potent, non-invasive diagnostic markers for different diseases including cancers, such as different types of lymphomas. Fang et al compared the level of 7 miRNA in serum of DLBCL patients to healthy controls using qRT-PCR showing 4 of them (miR-15a, miR-16, miR-29c and miR-155) to be significantly increased while one (miR-34a) was decreased [16]. Yuan et al correlated miRNA expression levels between serum and FFPE tissue, and analyzed the expression levels of eight miRNAs in DLBCL patients prior to treatment, showing a significant association of these miRNA between serum and matching tumor biopsy samples [17]. Jones et al performed microarray profiling of human miRNAs in 14 cHL primary tissues and 8 healthy lymph nodes and revealed a number of new disease node—associated miRNAs, including miR-494 and miR-1973. Using quantitative real-time PCR (qRT-PCR), they tested the utility of plasma miR-494, miR-1973, miR-155, miR-21 and miR-16 as disease response biomarkers in an independent prospective cohort of 42 patients with cHL and 20 healthy participants. Levels of miR-494, miR-1973 and miR-21 were higher in patients’ plasma compared to controls’ plasma, returning to normal at remission [18]. Van Eijndhoven et al claimed that tumoral miRNA are shed into the plasma via EVs and thus evaluated miRNA expression profile in plasma isolated EV showing enriched levels of miR-24, miR-127, miR-21, miR155 and let-7a in HL patients compared to healthy subjects [19].

In search for sensitive biomarkers that could precede obvious clinical relapse, we aimed to identify a wider range of plasma miRNA that could distinguish between control and lymphoma patients and serve for further studies on therapy response and early relapse. Since we did not detect major differences in miRNA content between healthy subjects' whole plasma and EV fraction, we analyzed our patients' plasma without further fractionation.

## Materials and methods

### Subjects

All experiments were conducted with approval of the local Helsinki ethical Committee of the Hadassah Medical Center (No.: HMO-0043-11) and a written informed consent was obtained from all the subjects, no identifying information or images were used. All experiments were performed in accordance with relevant guidelines and regulations. We recruited 20 healthy adult volunteers, 14 DLBCL patients and 11 Hodgkin lymphoma patients at disease or relapse diagnosis. Blood was drawn from each patient once, at screening, either when first diagnosed with lymphoma (de novo disease) or at diagnosis of relapse. Aiming for a high signal from patients' plasma, inclusion criteria required high LDH at diagnosis or a tumor mass above 5 cm. Clinical parameters were prospectively recorded. International Prognostic Index (age >60, ECOG performance status over 2, stage III-IV, extranodal site, LDH above the upper normal limit) was calculated for all DLBCL patients, who were accordingly sub-divided into four risk groups (low, low-intermediate, high-intermediate and high). HL patients were defined as early-stage disease according to the Southwest Oncology Group (SWOG) and Cancer and Leukemia Group B (CALGB) previously published definition (Ann Arbor stages I or II without any B symptoms, infra diaphragmatic presentations, or mediastinal masses greater than one-third the maximum thoracic diameter); the rest were defined as advanced disease.

### Plasma isolation

Ten mL of blood were obtained by venipuncture using a 21G needle and collected in EDTA containing tubes. Blood was immediately processed by centrifugation at 1,000g for 5 min at room temperature to separate plasma. The isolated plasma was transferred to 1.5 ml Eppendorf tubes and centrifuged at 12,000g for 45 min at 4°C. Supernatant was transferred to a 0.22 μm membrane pore size centrifuge filter (Corning^®^ Costar^®^ Spin-X^®^ centrifuge tube filters) and spun at 15,000g for 2 minutes. Specimens were then aliquoted into 1.5 ml Eppendorf tubes, 500 μl in each tube, and frozen at -80°C until further analysis.

For isolation of plasma exosomes, samples were thawed and subjected to two sequential ultracentrifugations at 100,000g. The second centrifugation pellet was resuspended in 50 μl of saline for RNA extraction.

### Total RNA extraction and quantification

RNA was extracted from 425 μl of plasma or from exosomes extracted from the same volume of plasma. Samples were first incubated with proteinase K. Organic extraction was then preformed to remove hydrophobic peptide fragments, using a homemade reagent containing guanidinium isothiocyanate (GITC), phenol, citric acid, NaOH and sarcosyl [20]. Following chloroform extraction, isopropanol was added to the aqueous phase. To purify the RNA from the aqueous-isopropanol mixture we bound to commercial RNeasy MinElute columns. The columns then underwent repeated washes under vacuum, followed by elution with double distilled water. Total RNA was quantified using Qubit 2.0 (Thermo Fisher Scientific Inc.).

### Small RNA sequencing

Total RNA was subjected to in-house multiplexed small RNA cDNA library preparation, which entailed ligation of barcoded 3′ adapters to 20 different samples, pooling of samples, ligation of a 5′ adapter, reverse transcription and polymerase chain reaction (PCR), as previously described [13], with modifications allowing multiplexing of several 20-sample libraries on a single HiSeq lane, namely, 40–100 small RNA samples per lane [21]. Libraries were sequenced on an Illumina HiSeq sequencer, and the information obtained was analyzed by an automated computer pipeline to decode and annotate small RNA reads [22]. Of all known human miRNA, 159 unique miRNA were detected with at least 1 read in at least 25% of samples.

### Statistical analysis

Statistical procedures on count data were based on the ‘DESeq2’ R/Bioconductor package for analysis of differential expression in RNA sequencing experiments [23], as previously described [24], and complemented by parallel calculations with ‘edgeR’ [25] and ‘limma’ [26]. We used ‘cancerclass’, a machine learning R/Bioconductor package, for development and validation of classification tests from the high-dimensional molecular data. The protocol of cancerclass uses simple classification methods, and includes validation and visualization of classification results. The protocol starts with feature selection by a filtering step. Then, a predictor is constructed using the nearest centroid method. The accuracy of the predictor is evaluated using training and test set validation [27], leave-one-out cross-validation or in a multiple random validation protocol. Survival analyses were conducted using ‘samr’ [28] (SAMseq command) and ‘survival’ [29] R packages. Plots were generated with ‘ggplot2’.

### Target prediction and pathway analysis

Ingenuity pathway analysis (IPA) software (Ingenuity Systems, Redwood City, CA) was applied to analyze the canonical pathways networks, and biological functions of the differentially expressed miRNA in the DLBCL and HL patients group, while filtering for high confidence prediction and experimental targets. The IPA software is based on computational algorithms of the connectivity from information obtained within the IPA (IPA, Qiagen, http://www.ingenuity.com). In order to predict the activation state of canonical pathways that were found to be enriched in these two datasets, an artificial fold change value was assigned to the targets, a positive one if the microRNA is down-regulated and an identical negative one, if the microRNA is upregulated. The two datasets were subjected to IPA core analysis. These assignments follow the assumption that microRNAs and their targets have opposite directions of expression, i.e. that an up-regulation of a microRNA causes the inhibition of its downstream targets and that the down-regulation of a microRNA causes the activation of downstream targets. This procedure uses the fact that the z-score that is calculated in IPA core analysis to predict the activation state of canonical pathways, only takes the direction of expression (i.e. up/down-regulation) into account, but not the intensities.

## Results

### Study participants' characteristics

Twenty healthy volunteers at a median age of 37 years participated in the control arm (11 men and 9 women). Patients were enrolled before starting therapy. Fourteen DLBCL patients were studied, all of them with de novo disease. Median age was 63.5 years; 9 men and 5 women. IPI score was low, low intermediate, high-intermediate and high in 4, 3, 3, 4 patients respectively. All DLBCL patients had a tumor mass larger than 5 cm and 9 had higher than normal serum LDH levels. Eleven classical HL (cHL) patients were recruited, of them 10 with de novo disease and 1 with relapsed disease. Median age was 27 years; 8 men and 3 women. Two were defined as having early stage disease and 9 had advanced disease. All cHL patients had a tumor mass larger than 5 cm and 2 had LDH levels higher than normal (Table 1).

**Table 1:** Clinical characteristics of study participants.

	DLBCL	cHL	Healthy
Sex, F:M	5:9	3:8	9:11
Age, median (range)	64 (34–85)	27 (18–43)	37 (26–63)
Phase (de novo/relapse)	14:0	10:1	n/a
Mass >5 cm	100%	100%	n/a
IPI score (L:LI:HI:H)	4:3:3:4	n/a	n/a
HL staging, E:A	n/a	2:9	n/a

DLBCL, diffuse large B-cell lymphoma; cHL, classic-type Hodgkin lymphoma; IPI, international prognostic index; E:A, early:late.

### miRNA profiles in healthy volunteers

**Fig 1:**
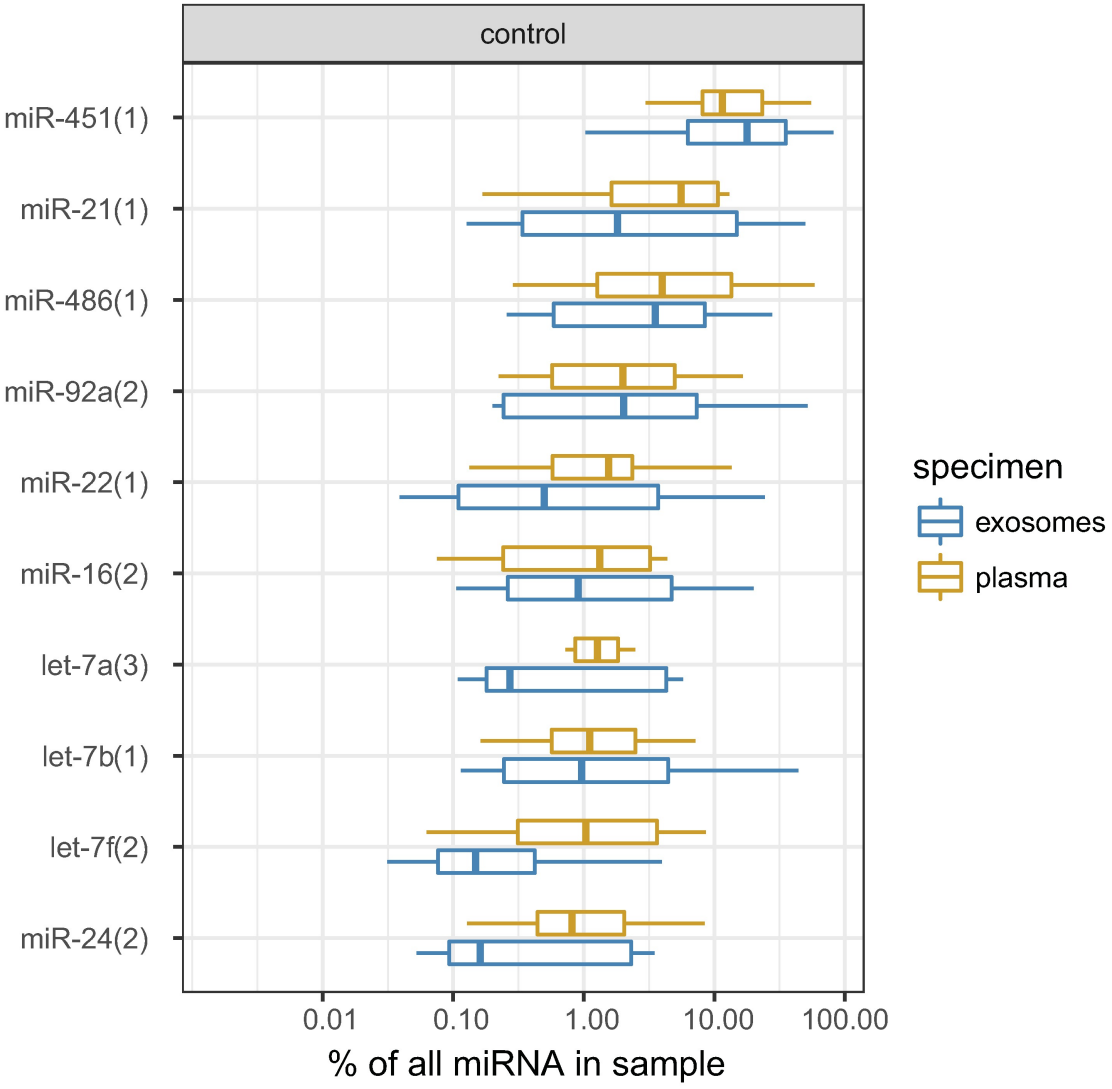
Box plots showing levels of top 10 miRNA in healthy controls' plasma and corresponding levels in paired exosome preparations. Statistically differences between plasma and exosomes are not significant, as inferred from a DESeq2 analysis presented in Table A in S2 File.

Small RNA libraries prepared from whole plasma or plasma exosome preparations were enriched with miRNA, which composed 22.3% of the sequenced reads (25^th^-75^th^ percentiles 15.0%-35.8%). Other frequently detected small RNAs were mRNA (median 10.6%, 6.6%-13.1%), rRNA (8.2%, 5.9%-11.9%), tRNA (1.4%, 1.0%-2.2%) and non-annotated RNA (36.3%, 27.8%-52.6%). The distribution of small RNA categories did not differ between whole plasma and exosome preparations (**Fig A** in S1 File and **Table A** in S2 File). miRNA profiling in healthy volunteers revealed that the top 10 whole plasma miRNA are miR-451, miR-486, miR-21, miR-92a, let-7a, miR-22, let-7b, miR-16, miR-24 and let-7f. Exosomal miRNA content was not significantly different for any single miRNA when compared with whole plasma (Fig 1, **Fig A** in S1 File and **Table A** in S2 File). Further analyses were thus based on unfractionated plasma specimens. Individual miRNA counts in all study samples, after aggregation of technical repeats and batch corrections, are provided in **Table A** in S2 File.

### miRNA in HL and DLBCL patients’ plasma compared to healthy volunteers

**Fig 2:**
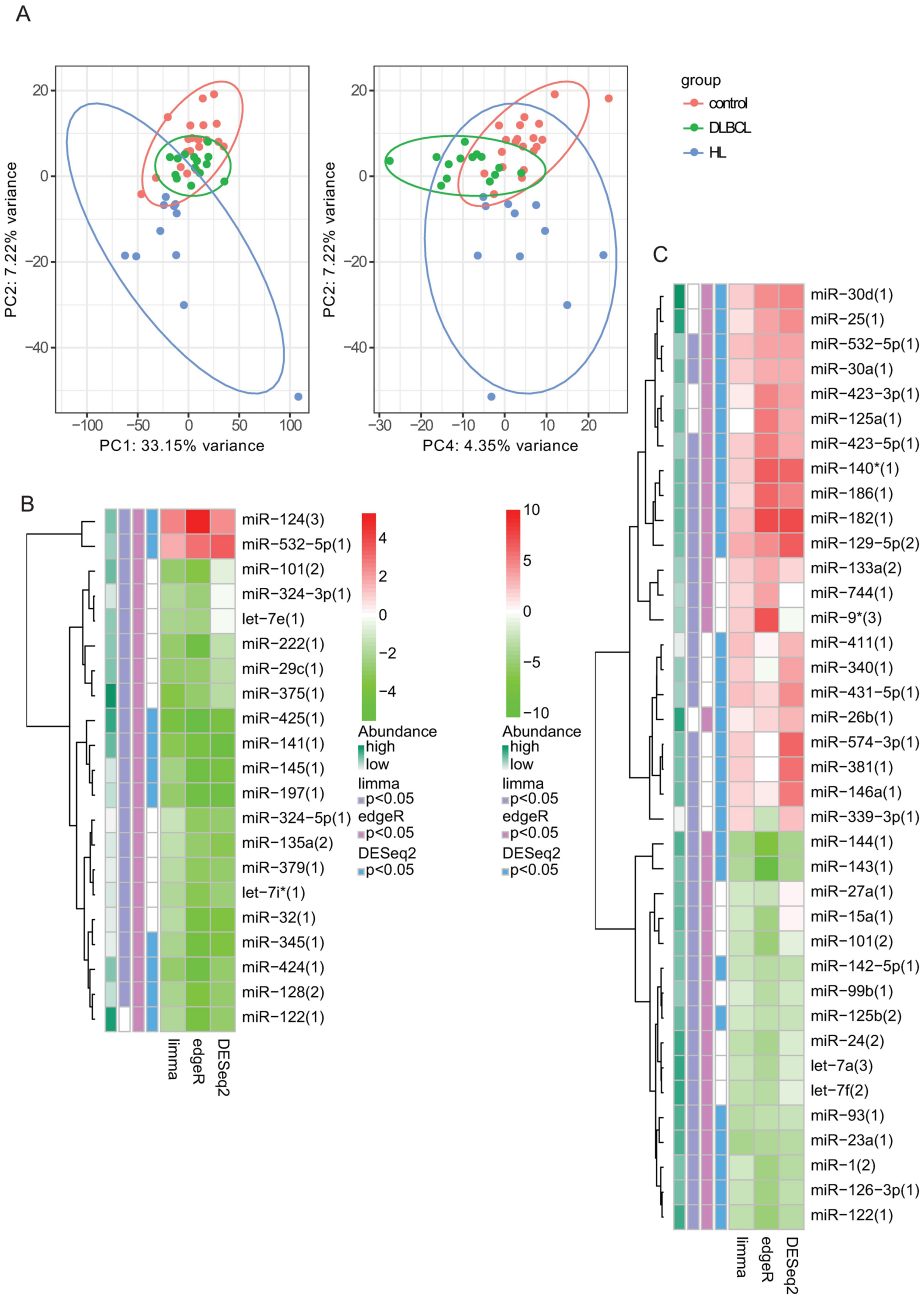
miRNA profiles in patients vs. controls. (**A**) Principal component (PC) analysis plots based on miRNA profiles; left panel—PC2 vs. PC1, right panel—PC2 vs. PC4. (**B&C**) Differentially expressed miRNA in DLBCL patients (**B**) or HL patients (**C**) compared to healthy controls according to three independent statistical approaches. Red shades represent upregulated miRNA while green shades represent downregulated miRNA. Row annotations (left to right) represent the overall abundance of each miRNA and the statistical significance according to limma, edgeR and DESeq2 approaches.

miRNA that were differentially expressed in patients’ plasma compared to controls are displayed in **Table B** in S2 File (sheets **1–6**). Principal component analysis based on the expression levels of all miRNA showed substantial separation of patients from controls (Fig 2A). miRNA that were significantly altered, according to at least 2 of 3 statistical approaches are shown in Fig 2 (panels **B** and **C**) and **Fig B** in S1 File. Among the most up-regulated miRNA were miR-124 and miR-532-5p in DLBCL patients and miR-182 and miR-140* in HL patients. Among the most down-regulated miRNA were miR-425 and miR-145 in DLBCL patients and miR-144 and miR-143 in HL patients. There was a significant correlation between the dysregulation noted in HL patients vs. controls and the dysregulation found in DLBCL patients vs. controls (**Fig B** in S1 File).

### Discrimination of patients from controls based on individual plasma miRNA

**Fig 3:**
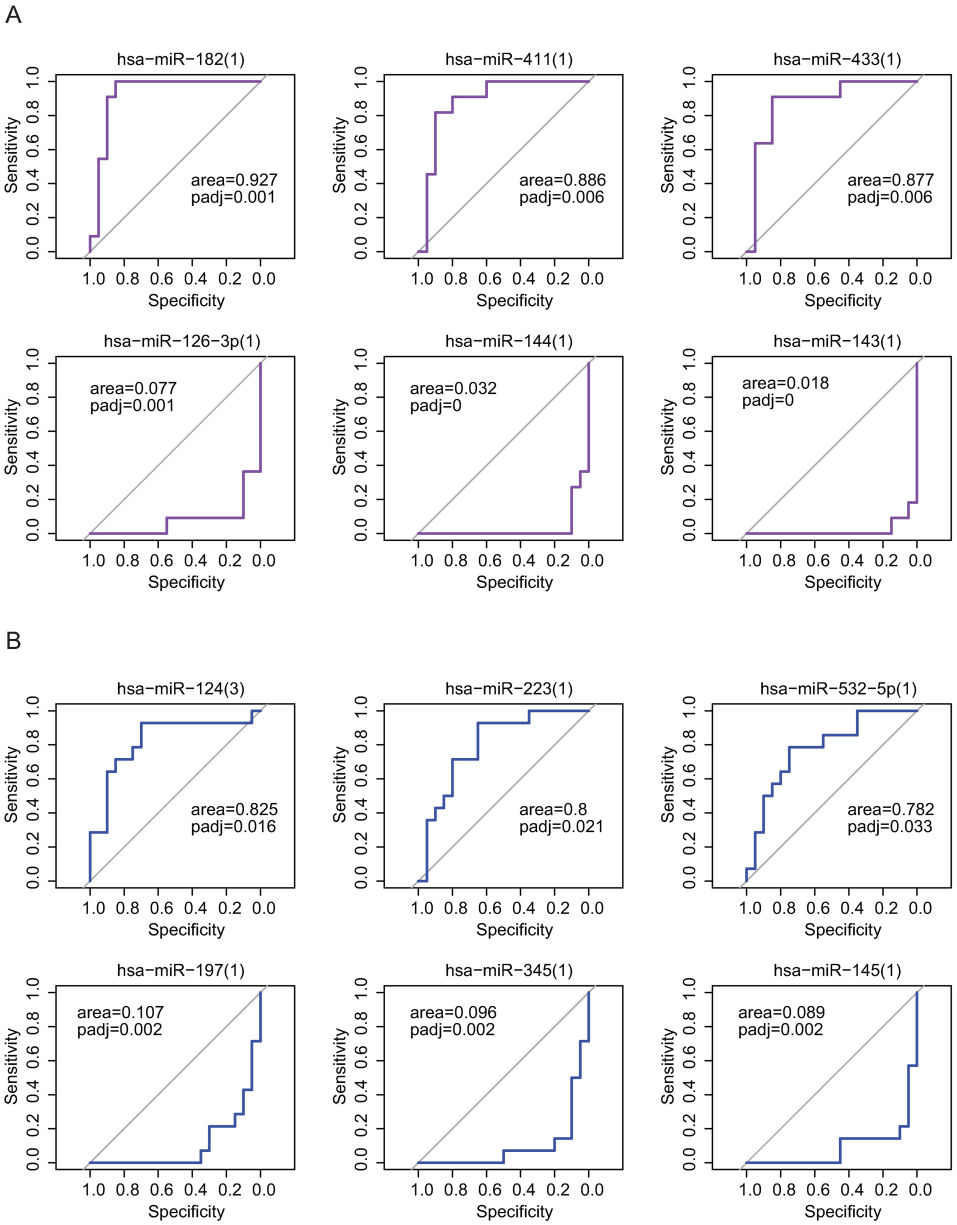
Receiver operating characteristics (ROC) curves describing discrimination of HL patients (A) or DLBCL patients (B) from controls with transformed levels of selected up-regulated and down-regulated miRNA.

Individual plasma miRNA provided variable power to discriminate patients from controls (**Table C** in S2 File, **Fig C** in S1 File). For example, the area under the ROC curve of miR-182 in HL vs. control subjects was 0.927 (adjusted p-value = 0.001, Fig 3A), while miR-223's area under the curve in DLBCL vs. control was 0.800 (adjusted p-value = 0.021, Fig 3B).

### Discrimination of patients from controls based on multiple miRNA

**Fig 4:**
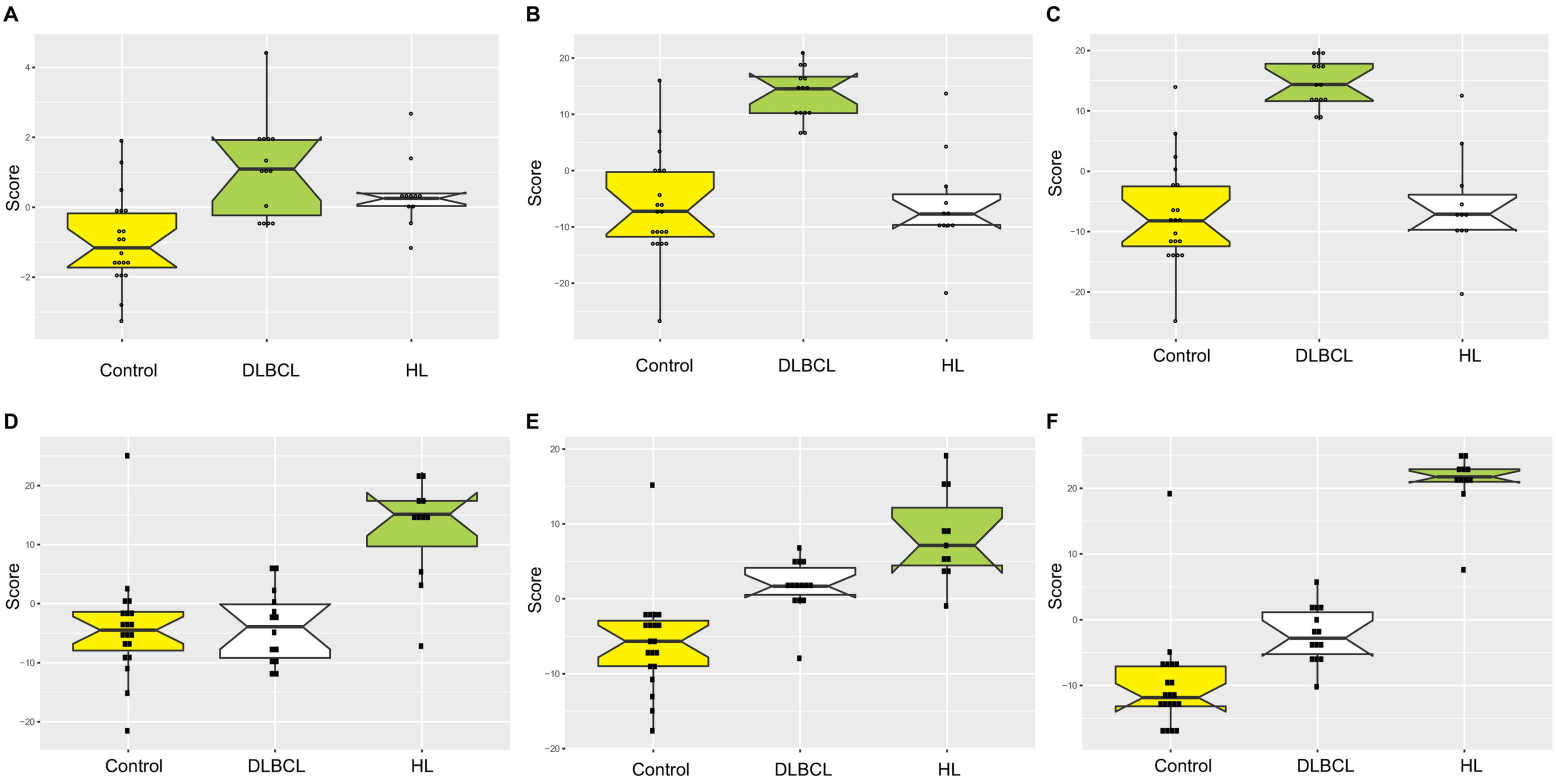
Box plots showing the distribution of composite scores based on the levels of differentially expressed miRNA between DLBCL patients and healthy volunteers (A-C) or between HL patients and healthy volunteers (D-F). Scores are composed of up-regulated miRNA (**A** and **D**), down-regulated miRNA (**B** and **E**) or all dysregulated miRNA (**C** and **F**) in the respective pairwise analysis; however, all 3 groups of patients are shown in all plots. The composite scores were calculated for each patient by standardizing miRNA levels (namely, scaling the average level of each miRNA to 0 and standard deviation of 1) and summing the respective miRNA. Upregulated miRNAs were added to the score sum, while downregulated miRNA were subtracted.

Composite scores based on all significantly up-regulated or down-regulated miRNA (depicted in Fig 2 and Table B in S2 File, sheets 1 and 2) were different across the groups of study participants (Table 2). However, when all differentially expressed miRNA (both up- and down-regulated) were included in the score, discrimination was greatest (Fig 4, and **Fig D** in S1 File).

**Table 2:** Discrimination value of up and down regulated plasma miRNA.

	AUC (c-statistic)	P-value
DLBCL vs. control, up-regulated miRNA	0.875	9.83e-05
DLBCL vs. control, down-regulated miRNA	0.957	3.91e-07
DLBCL vs. control, all dysregulated miRNA	0.979	4.31e-08
HL vs. control, up-regulated miRNA	0.891	0.000152
HL vs. control, down-regulated miRNA	0.959	2.29e-06
HL vs. control, all dysregulated miRNA	0.991	9.45e-08

The composite score was calculated for each patient by standardizing miRNA levels (namely, scaling the average level of each miRNA to 0 and standard deviation of 1) and summing the respective miRNA. Upregulated miRNA were added to the score sum, while downregulated miRNA were subtracted.

### Prediction and validation with machine learning

We used the ‘cancerclass’ R/Bioconductor package for classifier development and validation based on the miRNA profile data. The algorithm was applied separately for DLBCL vs. controls and HL vs. controls. **Fig E** in S1 File plots the importance of each miRNA in a multiple random validation protocol. The most influential miRNA are also listed in Table 3. Misclassification rate was dependent on the number of miRNA included in the predictor, with plateau at ~10% in both assessments (**Fig E** in S1 File).

**Table 3:** Top 10 influential miRNA for classification; inclusion rate in 200 iterations, each retaining 80 miRNAs.

DLBCL vs. control		HL vs. control	
miRNA	Inclusion rate	miRNA	Inclusion rate
miR-425	100%	miR-144	100%
miR-141	100%	miR-143	100%
miR-197	100%	miR-129-5p	98%
miR-145	100%	miR-182	96%
miR-345	100%	miR-411	96%
miR-200c	97%	miR-126-3p	93%
miR-324-5p	94%	miR-433	91%
let-7i*	94%	miR-23a	90%
miR-424	91%	miR-24	84%
miR-222	90%	miR-423-5p	82%

**Fig 5:**
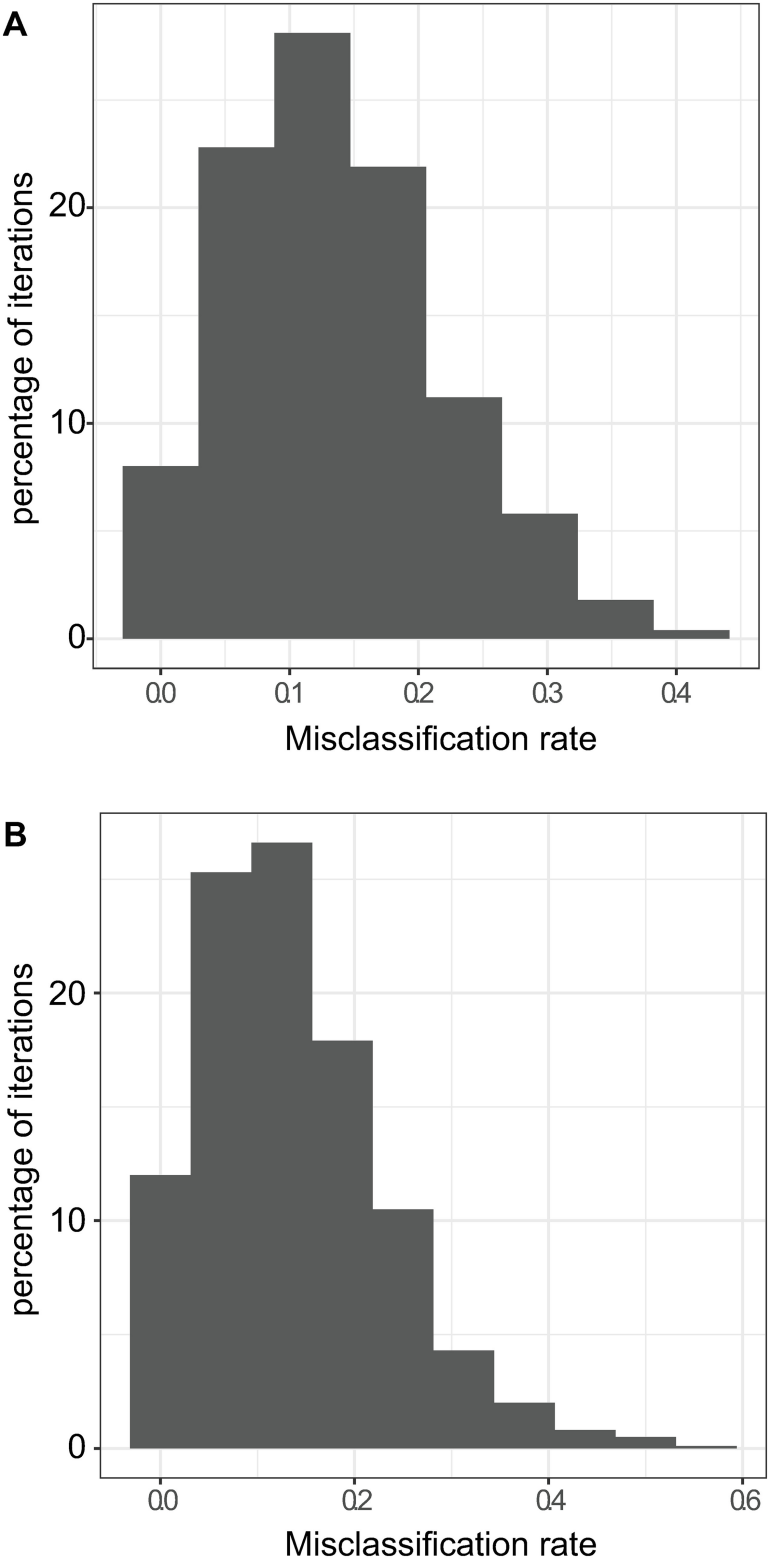
Distributions of misclassification rates within 1000 iterations of training and validation using 'cancerclass', performed on DLBCL patients' and healthy controls' miRNA profiles (A) or HL patients' and healthy controls' profiles (B).

Lastly, we trained the cancerclass protocol 1000 times on random 50% subsets of the samples and subsequently the classifier was tested on the rest of the samples (validation). Median misclassification rate of validation samples in DLBCL vs. controls was 11.8%, interquartile range (IQR) 5.9%-17.6% (Fig 5A). In HL vs. control validations, median misclassification rate was 12.5%, IQR 6.3%-18.8% (Fig 5B).

### Signaling pathway analysis

Ingenuity Pathway Analysis (IPA) was used to identify mRNAs targeted by the differentially expressed miRNA in the DLBCL and HL patients group and the biological processes in which they are involved. Looking at the most differentially abundant miRNA in each group we have created two datasets of the mRNA targets of miR-124 and miR-532-5p, miR-141, miR-145, miR-197, miR-345, miR-424, miR-128 and miR-122 for the DLBCL patient group and miR-25, miR-30a/d, miR-26b, miR-182, miR-186, miR-140* and miR-125a for the HL patient group. Using the high confidence experimental and predicted IPA database, in the DLBCL patient group mainly STAT3, IL-8, p13k/AKT and TGF-B signaling pathways were identified to be potentially up regulated, while PTEN and p53 pathways to be potentially down-regulated (**Fig F** in S1 File). In the HL patients group we identified mainly cAMP mediated pathway and p53 pathway to be potentially down-regulated (**Fig F** in S1 File).

### Survival analysis with miRNA

We evaluated the relationships of baseline plasma miRNA profiles with outcome, all-cause mortality, in lymphoma patients. Of 25 lymphoma patients, six died during a median follow-up of 4.2 years, range 0.9–5.3 (**Fig G** in S1 File). Age, sex and disease group (DLBCL vs. HL) did not significantly predict mortality (not shown). The potential of miRNA to predict mortality was explored by survival analysis with miRNA counts as predictors using ‘samr’. The Q-Q plot shown in **Fig G** in S1 File depicts the existence of miRNA that associate with all-cause mortality beyond chance associations expected due to multiple testing. Lastly, we evaluated the leading miRNA in the samr analysis individually using cox proportional hazards models; most were indeed significantly associated with mortality (**Fig G** in S1 File). Specifically, plasma levels above the median were associated with the following hazards ratios for mortality: miR-20b, 1.73 (p-value 0.0022); miR-1, 1.50 (p-value 0.0021); miR-93, 1.45 (p-value 0.0286); miR-20a, 1.40 (p-value 0.0246); miR-128, 1.38 (p-value 0.0345); miR-106b, 1.31 (p-value 0.0469); miR-106a, 1.25 (p-value 0.0401) and miR-200c, 0.66 (p-value 0.0274).

## Discussion

Early detection and treatment of relapsed lymphoma can improve response and survival. Current tools lack sensitivity and specificity for detection of early relapsed disease, while being cumbersome and expensive [30]. For this reason, sensitive, non-invasive disease biomarkers are needed. Many studies of circulating miRNAs in cancer demonstrated a non-invasive accurate detective or prognostic potential using a combination of miRNA expression levels, and the diagnosis of cancer even at early stages. These short RNA species are robust, survive harsh treatment and prolonged storage conditions and may be extracted from blood [31]. Matsuzaki and Ochiya identified over 200 high quality published studies that provide strong evidence that circulating miRNAs and EV-associated components are promising biomarkers for cancer diagnosis and prognosis. Nevertheless, endogenous controls to normalize circulating miRNA levels have not been standardized, which leads to conflicting results across studies [32]. MicroRNA profiling via microarrays or NGS offers high-throughput with respect to assaying the expression levels of hundreds to thousands of miRNAs or miRNA variants in a single experiment, which is especially useful in early biomarker discovery efforts. NGS further enables detection of novel miRNAs and the precise identification of miRNA sequences increasing the amount of sequence output per run, with improved computational accuracy compared to traditional sequencing methods. In miRNA sequencing (miRNA-seq), the number of reads obtained per miRNA directly correlates with the abundance of the miRNA in the sample, which is linked to its relative expression level [33]. Serum biomarkers of lymphoma activity for diagnosis, prognosis, and therapy monitoring are of great clinical interest in the last decade. There are opportunities to evaluate levels of natural serum constituents, tumor produced enzymes, or even nucleic acids released from tumors that may represent dysregulated tumor drivers. While the initial diagnosis requires tissue sampling for clinical monitoring, robust biomarkers can help diagnosis, determination of clinical remission with greater sensitivity for residual disease than manual palpation or other clinical evaluations, or detection of disease progression prior to clinically detectable evidence of that event. In search for sensitive robust biomarkers that could precede obvious clinical relapse, we aimed to identify a wider range of plasma miRNA that could distinguish between control and lymphoma patients and serve for further studies on therapy response and early relapse. Unlike prior works, we did not restrict our assessment to tumor-associated miRNA, but aimed to look at the entire miRNA profile of lymphoma patients compared to healthy volunteers.

miRNA profiling in our healthy volunteers revealed that the top 10 whole plasma miRNAs are miR-451, miR-486, miR-21, miR-92a, let-7a, miR-22, let-7f, miR-16, miR-24 and let-7b. This is in agreement with previous reports [12, 14, 34]. The two most abundant miRNA were: miR-451, a Dicer independent miRNA comprising ∼50% of the miRNA content in red blood cells; and miR-486, transcribed from an intron in the ankyrin1gene (ANK1) expressed in endothelial cells, erythrocyte precursors and skeletal muscle[14].

We identified in DLBCL patients increased plasma levels of miR-124 and miR-532-5p, while miR-425, miR-141, miR-145, miR-197, miR-345, miR-424, miR-128 and miR-122 were down-regulated. Discrepancy between our results and those published by qRT-PCR analysis is in part due to the latter examining tumor overexpressed miRNA while we investigated the entire plasma mirnome, thus allowing many other possibly significant miRNA to be discovered [16, 17]. Ingenuity Pathway Analysis (IPA) was used to identify mRNAs targeted by these miRNAs and the biological processes in which they are involved. We have found potential up-regulation of the STAT3, IL8, p13k/AKT and TGF-B signaling pathways, while potential down-regulation of the PTEN and p53 pathways. PTEN is a ubiquitous tumor suppressor phosphatase known to inhibit pi3k/AKT which plays an important role in normal and malignant B cell biology affecting BCR signaling and downstream pathways affecting proliferation, differentiation and survival; pi3k/AKT serves as an attractive target in lymphoma therapy [35]. Serum IL-8 was found to be higher in DLBCL patients than in control subjects. While elevated levels of IL-8 correlated with more adverse disease features, lower response to therapy but did not affect overall survival [36].

We have shown that NGS of plasma miRNA may identify a set of miRNA that can serve as biological markers for relapsed lymphoma. Using next generation small RNA sequencing of patients' plasma, we have detected miRNA that were significantly altered between patients and controls. This is to our knowledge the first publication on deep sequencing of miRNA in plasma of DLBCL patients. Lawrie et al reported on levels of 3 different miRNAs analyzed by qRT-PCR [15] while Fang et al compared the levels of 7 miRNA in serum of DLBCL patients to healthy controls using qRT-PCR [16]. Yuan et al correlated qRT-PCR miRNA expression levels between serum and FFPE tissue, and analyzed the expression levels of eight miRNAs in DLBCL patients prior to treatment.

In our HL group, we identified plasma miR-25, miR-30d, miR-26b, miR-182, miR-186, miR-140*, miR-30a and miR-125a to be up regulated compared to controls, while miR-23a, miR-122, miR-93 and miR-144 were down-regulated. Our results cannot be directly compared to those in the literature since others have looked either at specific HL miRNA expression or at expression profiles of plasma sub-fractions (e.g. protein-bound, EVs isolated with size-exclusion chromatography) [11, 16, 18]. Using again IPA to identify mRNAs targeted by these miRNA, we have found mainly the cAMP mediated pathway and p53 pathway to be potentially down regulated. It has been shown that p53 inactivation/dysfunction alters the immune landscape of the tumor microenvironment (TME) towards pro-tumor inflammation [37], while cAMP is a potent negative regulator of T cell receptor-mediated activation of effector T cells [38].

Evaluation of the relationships between baseline plasma miRNA profiles and outcome of our lymphoma patients reveled plasma levels of miR-20a, 20b, 93 and 106a 106b to be associated with a higher hazard ratio for mortality. The miR-17–92 cluster and its paralogs miR-106a-and miR-106b encode for 13 miRNAs that act as oncogenes [39, 40]. The miR-17–92 cluster has been shown to be over-expressed in systemic lymphomas [41–44] and clinical translational studies have demonstrated the association between the overexpression of miR-17–92 and shorter survival in nodal diffuse large B-cell lymphoma (DLBCL), primary cutaneous B cell lymphoma and mantle cell lymphoma [45–47]. MiR-93, which belongs to the miR-106b-25 cluster, is an oncomiR in many types of human cancers. The aberrant expression and dysfunction of miR-93 have been associated with tumor progression, metastasis, and poor prognosis in hepatocellular carcinoma, lung cancer, breast cancer, gastric and nasopharyngeal carcinoma [48]. All of these miRNAs have been shown to down regulate PTEN expression and upregulate the pi3k/AKT pathway [40, 48, 49].

Since it was speculated that miRNA in the circulation are commonly found enclosed in EVs protecting them from degradation by RNases, we compared healthy controls' plasma miRNA to plasma-derived EV miRNA content. We have found that our fractionated EV miRNA content was not significantly different for any single miRNA when compared with whole plasma. Unlike Van Eijndhoven et al who reported that Hodgkin lymphoma tissues secrete a mixed population of tumor-derived EVs that bring lymphoma-associated miRNAs into the circulation (where they are protected from degradation), we did not aim at evaluating specific tumor secreted miRNA but rather at any highly expressed miRNA [19]. We have chosen to evaluate whole plasma content, but farther evaluation of fractionated EV miRNA content in patients’ plasma compared to unfractionated samples is warranted. Another limitation of our study is our relatively small cohort of patients. A larger external cohort is needed to validate our results.

To date, biomarker discovery has many limitations, one of which is the lack of optimal normalization method for analysis of plasma RNAseq data. Like others, we used an equal plasma volume input allowing the comparison of miRNA expression level between patient and control samples [18, 19]. In conclusion, using NGS of circulating miRNA we have been able to identify a different set of overexpressed circulating miRNA that might serve as detectable biomarkers for relapsed lymphoma. Our results need to be validated by an external cohort of patient samples processed by our laboratory using next generation sequencing or by performing qRT-PCR of the specific up-regulated/down-regulated miRNA expression levels and by sequential sampling of circulating miRNA at diagnosis and relapse settings. These are parts of our future research plans.

## Supporting information

S1 File **Fig A.** (**A**) Box plots depicting the levels of different small RNA categories in plasma and exosome preparations in control subjects and DLBCL patients. (**B**) Box plots showing levels of top 10 miRNA (as determined in healthy controls' plasma) in DLBCL patients' plasma and corresponding levels in the paired exosome preparations. **Fig B.** Levels of differentially expressed miRNA (according to at least two statistical approaches) in DLBCL patients vs. healthy controls (**A**) or HL patients vs. healthy controls (**B**). (**C**) The log_2_ fold-change values in the HL vs. control comparison were plotted against the log_2_ fold-change values in the DLBCL vs. control comparison. **Fig C.** Empirical cumulative distribution function (eCDF) plots of area under the ROC curve values discriminating HL patients (**left**) or DLBCL patients (**right**) from controls applying 3 different count transformations; logCPM (edgeR), voom (limma) and vst (DESeq2). **Fig D.** Conditional density plots are illustrating the conditional distribution of the group variable (control, DLBCL, HL) over the values of the miRNA scores. miRNA scores were derived from differential expression analyses comparing HL patients to controls (left 2 panels) or DLBCL vs. controls (right 2 panels), as in Fig 4 and Table 2. Scores are comprised of upregulated miRNA (top panel), downregulated miRNA (middle panel) or all dysregulated miRNA (bottom panel). **Fig E.** The ‘cancerclass’ package was applied upon our miRNA profile data to obtain DLBCL vs. control classifiers (**A**&**B**) and HL vs. controls classifiers (**C**&**D**). Misclassification rate was dependent on the number of miRNA included in the predictor (panels **A** and **C**). The classification importance of each miRNA is depicted in panels **B** and **D**, as the percentage of repetitions in which the miRNA was included in the classifier. **Fig F.** Canonical pathways that are found to be enriched (FDR< = 0.05) in the datasets of mRNA targets of increased plasma miRNAs in DLBCL (**A**) and HL (**B**) patients: Each column represents an enriched IPA canonical pathway. The height of the column shows the negative log (Benjamini-Hochberg corrected p-value) of the canonical pathway. The orange line represents the ratio between the number of genes in our datasets and the total number of genes that are known to participate in that canonical pathway. Each column is colored according to its IPA z-score value, orange/blue for positive/negative z-score (predicting up/down-regulation of the canonical pathway). Gray columns represent a z-score that cannot be calculated due to a lack of knowledge. **Fig G.** (**A**) Kaplan-Meier plot showing 5-year survival of ~75% among all lymphoma patients. The dashed lines denote 95% confidence intervals. (**B**) A Q-Q plot based on survival-type ‘samr’ results, that shows expected vs. observed association scores of miRNA with mortality. Circles in upper-right represent plasma miRNA associated with increased mortality, while lower-left miRNA are linked with reduced mortality. Red circles represent miRNA with low q-values, namely significance withstanding adjustment for multiple testing. (**C**) Survival plots with individual miRNA as predictors of mortality. miRNA were chosen based on the samr analysis (A), and were used as predictors in Kaplan-Meier plots as categorical variables with cutoffs at the median levels.
(PDF)

S2 File **Table A.** (**A**) DESeq2 results displaying differential abundance of small RNA categories in exosome preparations compared to matched healthy controls' plasma samples. (**B**) DESeq2 results displaying differential abundance of miRNAs in exosome preparations compared to matched healthy controls' plasma samples. (**C**) Batch-corrected individual miRNA counts in all study samples (technical repeats aggregated). Table available at https://goo.gl/5G8lco. **Table B.** DESeq2 (sheets **1** and **2**), voom/limma (sheets **3** and **4**) and edgeR (sheets **5** and **6**) results displaying differential abundance of miRNA in DLBCL patients' plasma compared to healthy controls' plasma (sheets **1**, **3**, **5**) and HL patients' plasma compared to healthy controls’ plasma (sheets **2**, **4**, **6**). Table available at https://goo.gl/5G8lco. **Table C.** Area under the receiver operating characteristics (ROC) curves for discrimination of DLBCL patients (**A**) or HL patients (**B**) from controls according to voom-transformed plasma miRNA counts. Table available at https://goo.gl/5G8lco.
(DOCX)
