# Supplementary material for: Plasma microRNA profiling: Exploring better biomarkers for lymphoma surveillance
Source: PLoS One. 2017 Nov 13;12(11):e0187722. doi: 10.1371/journal.pone.0187722 (PMC5683633; doi:10.1371/journal.pone.0187722)
Supplement: S1 File — Fig A. (A) Box plots depicting the levels of different small RNA categories in plasma and exosome preparations in control subjects and DLBCL patients. (B) Box plots showing levels of top 10 miRNA (as determined in healthy controls' plasma) in DLBCL patients' plasma and corresponding levels in the paired exosome preparations. Fig B. Levels of differentially expressed miRNA (according to at least two statistical approaches) in DLBCL patients vs. healthy controls (A) or HL patients vs. healthy controls (B). (C) The log2 fold-change values in the HL vs. control comparison were plotted against the log2 fold-change values in the DLBCL vs. control comparison. Fig C. Empirical cumulative distribution function (eCDF) plots of area under the ROC curve values discriminating HL patients (left) or DLBCL patients (right) from controls applying 3 different count transformations; logCPM (edgeR), voom (limma) and vst (DESeq2). Fig D. Conditional density plots are illustrating the conditional distribution of the group variable (control, DLBCL, HL) over the values of the miRNA scores. miRNA scores were derived from differential expression analyses comparing HL patients to controls (left 2 panels) or DLBCL vs. controls (right 2 panels), as in Fig 4 and Table 2. Scores are comprised of upregulated miRNA (top panel), downregulated miRNA (middle panel) or all dysregulated miRNA (bottom panel). Fig E. The ‘cancerclass’ package was applied upon our miRNA profile data to obtain DLBCL vs. control classifiers (A&B) and HL vs. controls classifiers (C&D). Misclassification rate was dependent on the number of miRNA included in the predictor (panels A and C). The classification importance of each miRNA is depicted in panels B and D, as the percentage of repetitions in which the miRNA was included in the classifier. Fig F. Canonical pathways that are found to be enriched (FDR< = 0.05) in the datasets of mRNA targets of increased plasma miRNAs in DLBCL (A) and HL (B) patients: Each column represents [file pone.0187722.s001.pdf]

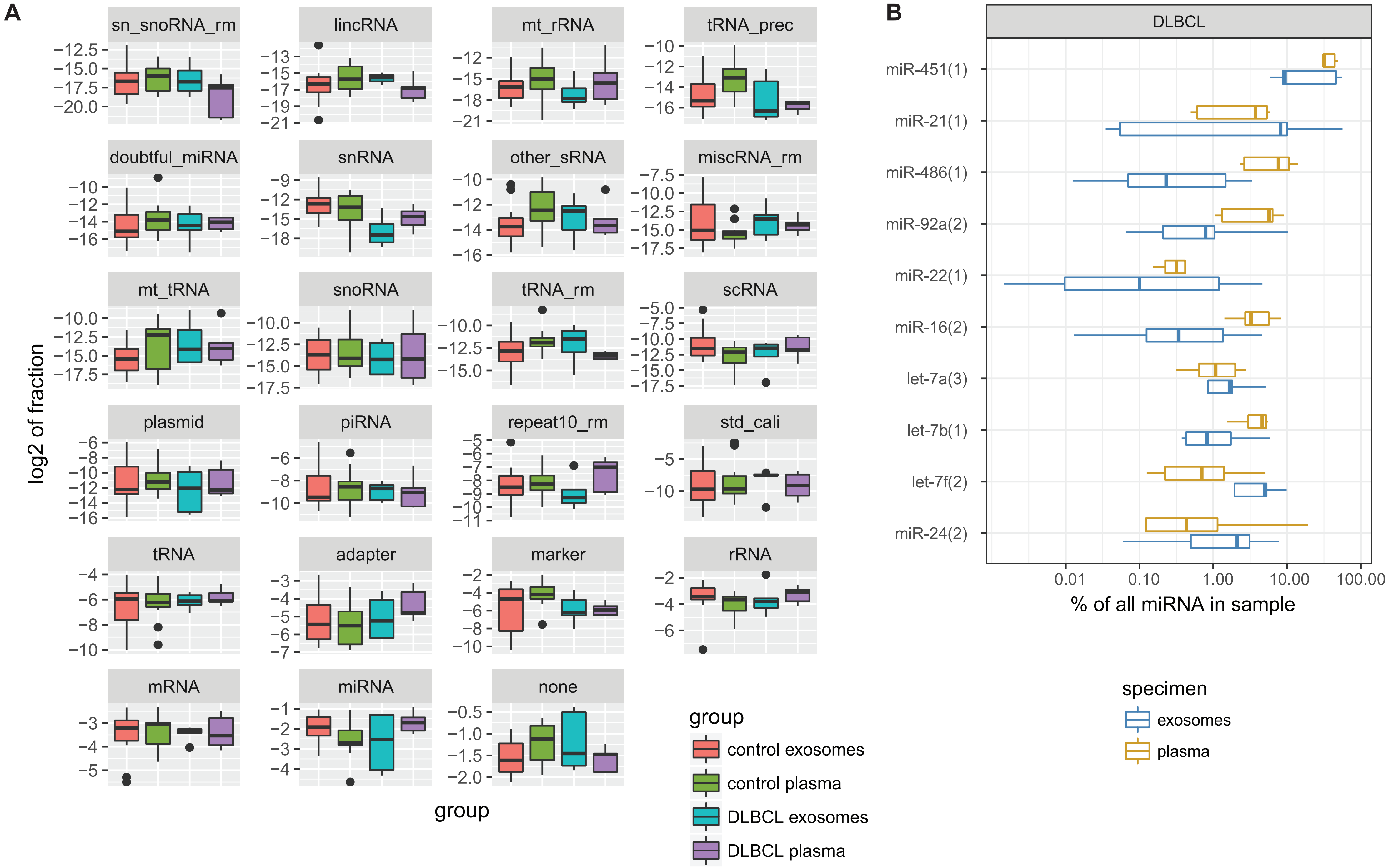

Figure A: (A) Box plots depicting the levels of different small RNA categories in plasma and exosome preparations in control subjects and DLBCL patients. (B) Box plots showing levels of top 10 miRNA (as determined in healthy controls' plasma) in DLBCL patients' plasma and corresponding levels in the paired exosome preparations

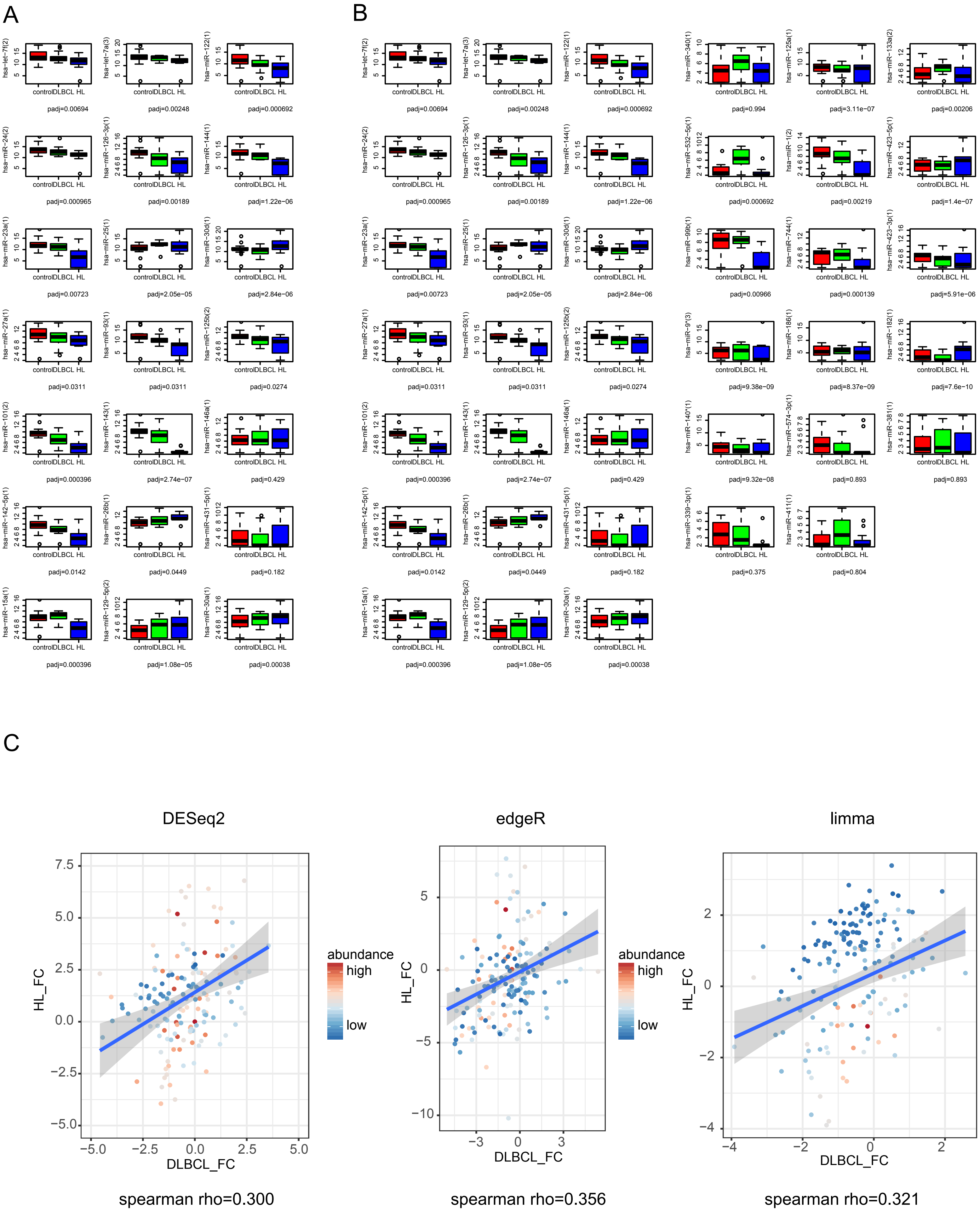

Figure B: Levels of differentially expressed miRNA (according to at least two statistical approaches) in DLBCL patients vs. healthy controls (A) or HL patients vs. healthy controls (B). (C) The log2 fold-change values in the HL vs. control comparison were plotted against the log2 fold-change values in the DLBCL vs. control comparison.

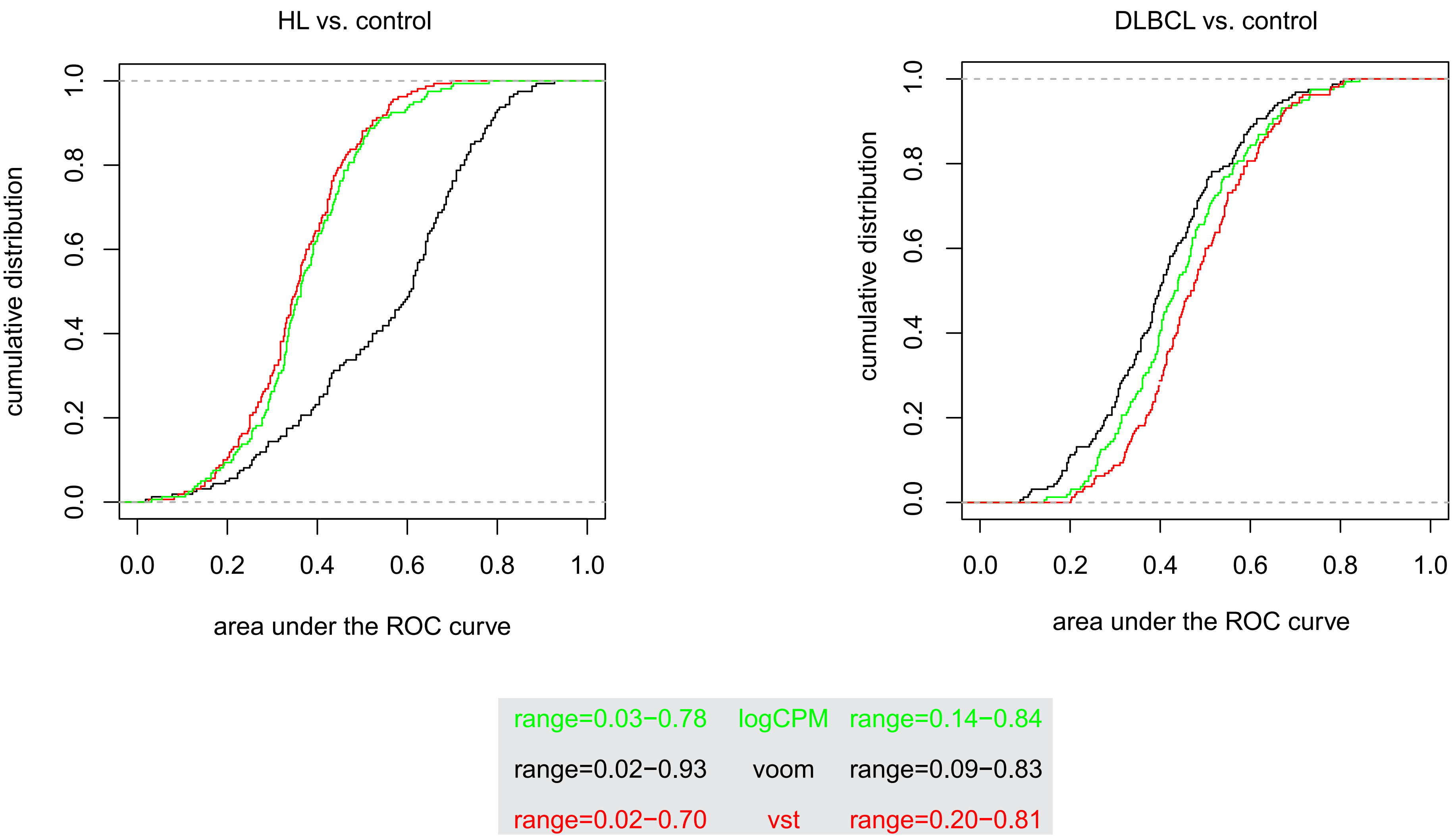

Figure C: Empirical cumulative distribution function (eCDF) plots of area under the ROC curve values discriminating HL patients (left) or DLBCL patients (right) from controls applying 3 different count transformations; logCPM (edgeR), voom (limma) and vst (DESeq2).

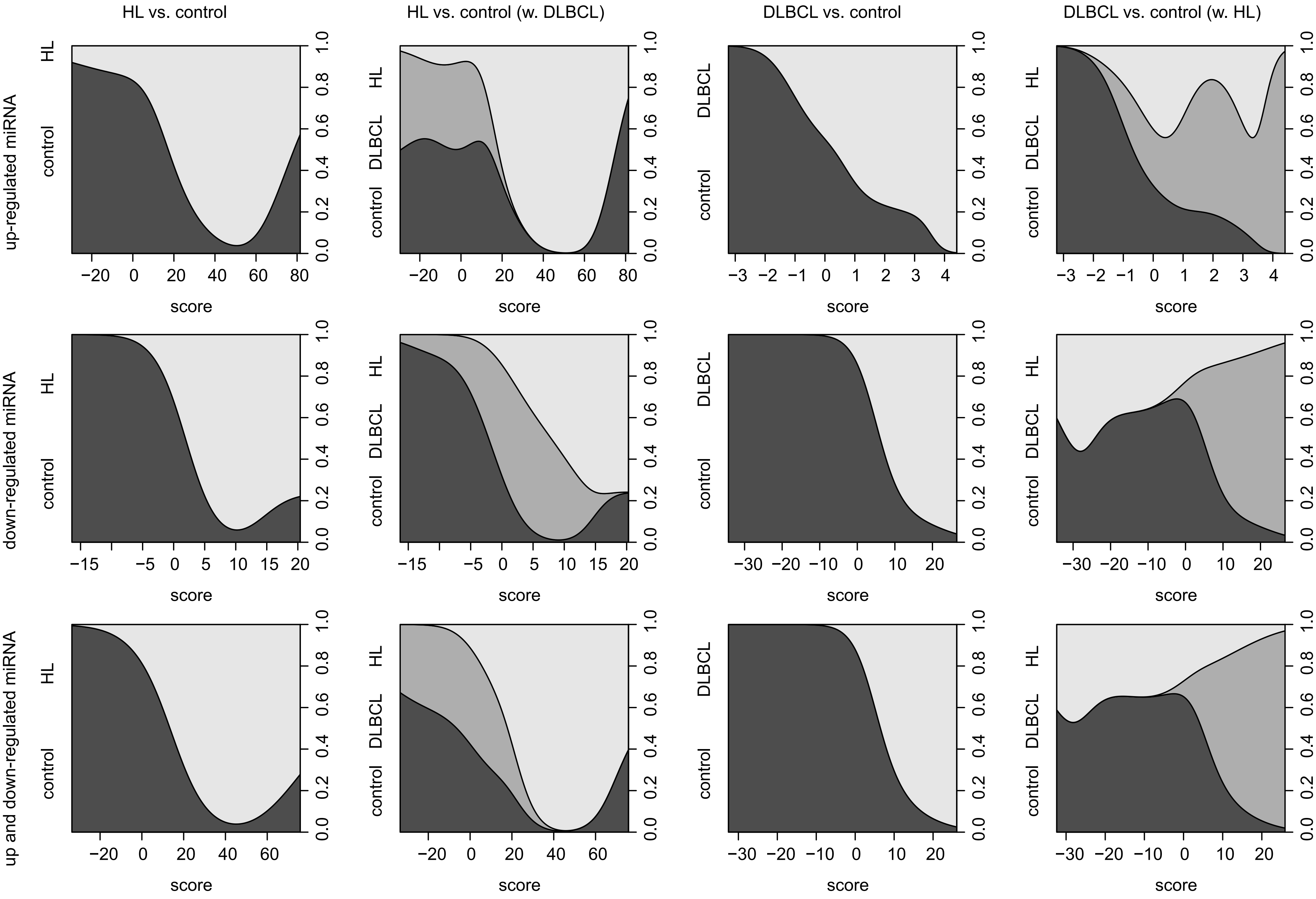

Figure D: Conditional density plots are illustrating the conditional distribution of the group variable (control, DLBCL, HL) over the values of the miRNA scores. miRNA scores were derived from differential expression analyses comparing HL patients to controls (left 2 panels) or DLBCL vs. controls (right 2 panels), as in Figure 4 and Table 2. Scores are comprised of upregulated miRNA (top panel), downregulated miRNA (middle panel) or all dysregulated miRNA (bottom panel).

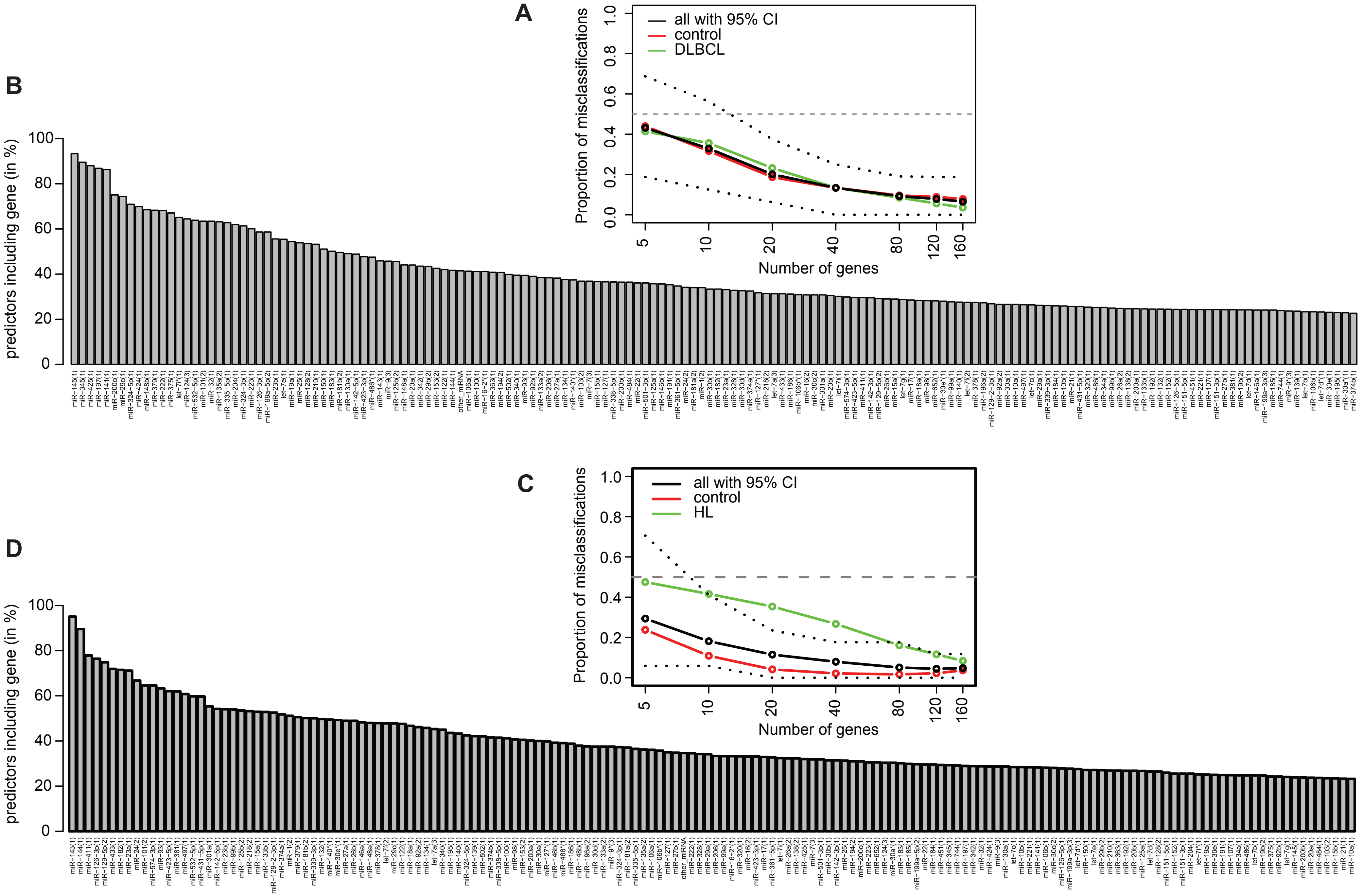

Figure E: The ‘cancerclass’ package was applied upon our miRNA profile data to obtain DLBCL vs. control classifiers (A&B) and HL vs. controls classifiers (C&D). Misclassification rate was dependent on the number of miRNA included in the predictor (panels A and C). The classification importance of each miRNA is depicted in panels B and D, as the percentage of repetitions in which the miRNA was included in the classifier.

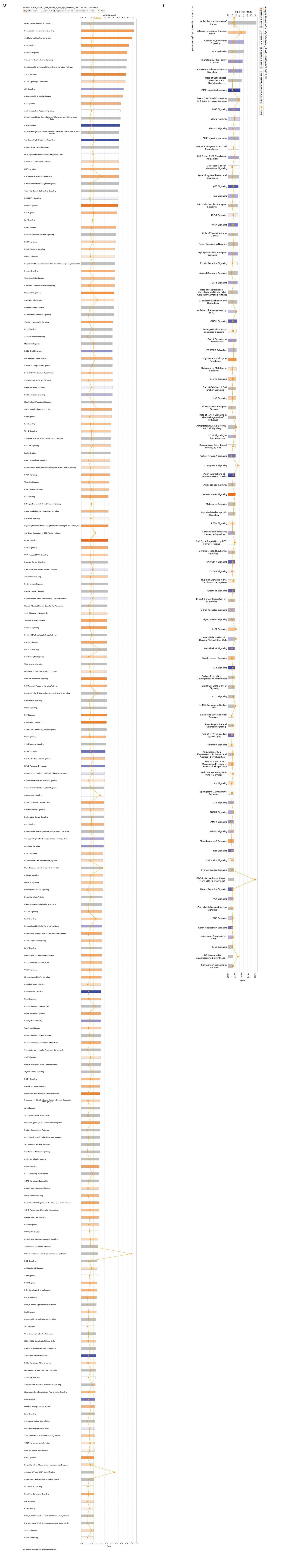

Figure F: Canonical pathways that are found to be enriched (FDR<=0.05) in the datasets of mRNA targets of increased plasma miRNAs in DLBCL (A) and HL (B) patients: Each column represents an enriched IPA canonical pathway. The height of the column shows the negative log (Benjamini-Hochberg corrected p-value) of the canonical pathway. The orange line represents the ratio between the number of genes in our datasets and the total number of genes that are known to participate in that canonical pathway. Each column is colored according to its IPA z-score value, orange/blue for positive/negative z-score (predicting up/down-regulation of the canonical pathway). Gray columns represent a z-score that cannot be calculated due to a lack of knowledge.

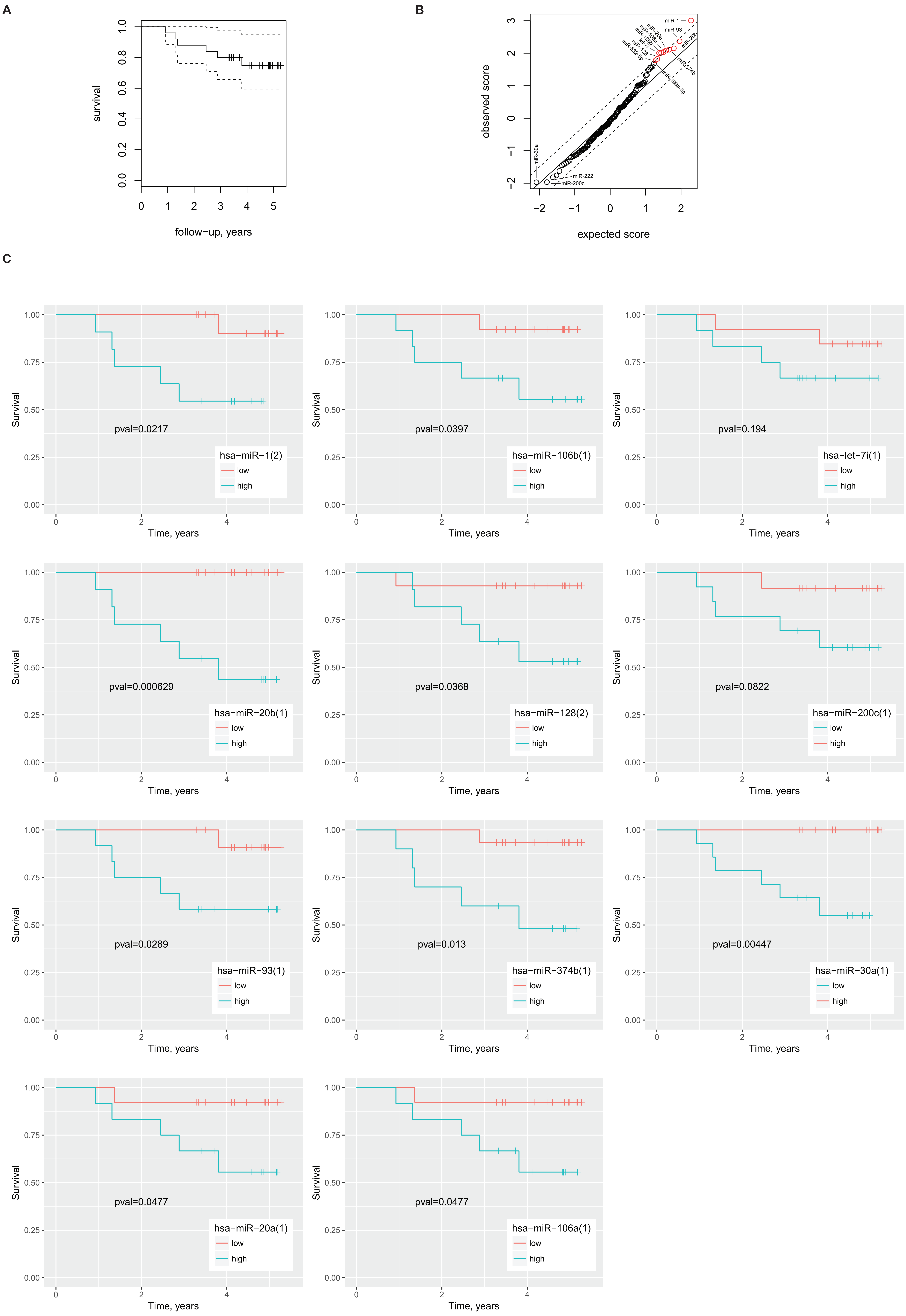

Figure G: (A) Kaplan-Meier plot showing 5-year survival of ~75% among all lymphoma patients. The dashed lines denote 95% confidence intervals.
(B) A Q-Q plot based on survival-type ‘samr’ results, that shows expected vs. observed association scores of miRNA with mortality.
Circles in upper-right represent plasma miRNA associated with increased mortality, while lower-left miRNA are linked with reduced mortality.
Red circles represent miRNA with low q-values, namely significance withstanding adjustment for multiple testing.
(C) Survival plots with individual miRNA as predictors of mortality. miRNA were chosen based on the samr analysis (A),
and were used as predictors in Kaplan-Meier plots as categorical variables with cutoffs at the median levels.
