# Supplementary material for: Plasma microRNA profiling: Exploring better biomarkers for lymphoma surveillance
Source: PLoS One. 2017 Nov 13;12(11):e0187722. doi: 10.1371/journal.pone.0187722 (PMC5683633; doi:10.1371/journal.pone.0187722)
Supplement: S2 File — Table A. (A) DESeq2 results displaying differential abundance of small RNA categories in exosome preparations compared to matched healthy controls' plasma samples. (B) DESeq2 results displaying differential abundance of miRNAs in exosome preparations compared to matched healthy controls' plasma samples. (C) Batch-corrected individual miRNA counts in all study samples (technical repeats aggregated). Table available at https://goo.gl/5G8lco. Table B. DESeq2 (sheets 1 and 2), voom/limma (sheets 3 and 4) and edgeR (sheets 5 and 6) results displaying differential abundance of miRNA in DLBCL patients' plasma compared to healthy controls' plasma (sheets 1, 3, 5) and HL patients' plasma compared to healthy controls’ plasma (sheets 2, 4, 6). Table available at https://goo.gl/5G8lco. Table C. Area under the receiver operating characteristics (ROC) curves for discrimination of DLBCL patients (A) or HL patients (B) from controls according to voom-transformed plasma miRNA counts. Table available at https://goo.gl/5G8lco. (DOCX) [file pone.0187722.s002.docx]

***Table A***

(**A**) DESeq2 results displaying differential abundance of small RNA categories in exosome preparations compared to matched healthy controls' plasma samples. (**B**) DESeq2 results displaying differential abundance of miRNAs in exosome preparations compared to matched healthy controls' plasma samples. (**C**) Batch-corrected individual miRNA counts in all study samples (technical repeats aggregated). The table is available at <https://goo.gl/5G8lco>.

***Table B***

DESeq2 (**A** and **B**), voom/limma (**C** and **D**) and edgeR (**E** and **F**) results displaying differential abundance of miRNA in DLBCL patients' plasma compared to healthy controls' plasma (**A**, **C**, **E**) and HL patients' plasma compared to healthy controls' plasma (**B**, **D**, **F**). The table is available at <https://goo.gl/5G8lco>.

***Table C***

Area under the receiver operating characteristics (ROC) curves for discrimination of DLBCL patients (**A**) or HL patients (**B**) from controls according to voom-transformed plasma miRNA counts. The table is available at <https://goo.gl/5G8lco>.
